# Exploring aflatoxin contamination and household-level exposure risk in diverse Indian food systems

**DOI:** 10.1371/journal.pone.0240565

**Published:** 2020-10-26

**Authors:** Anthony Wenndt, Hari Kishan Sudini, Prabhu Pingali, Rebecca Nelson

**Affiliations:** 1 Plant Pathology and Plant-Microbe Biology, School of Integrative Plant Sciences, Cornell University, Ithaca, New York, United States of America; 2 Tata Cornell Institute for Agriculture and Nutrition, Cornell University, Ithaca, New York, United States of America; 3 International Crops Research Institute for the Semi-Arid Tropics, Patancheru, Telangana, India; 4 Charles H. Dyson School of Applied Economics & Management, Cornell University, Ithaca, New York, United States of America; Ghent University, BELGIUM

## Abstract

The present study sought to identify household risk factors associated with aflatoxin contamination within and across diverse Indian food systems and to evaluate their utility in risk modeling. Samples (n = 595) of cereals, pulses, and oil seeds were collected from 160 households across four diverse districts of India and analyzed for aflatoxin B1 using enzyme-linked immunosorbent assay (ELISA). Demographic information, food and cropping systems, food management behaviors, and storage environments were profiled for each household. An aflatoxin detection risk index was developed based on household-level features and validated using a repeated 5-fold cross-validation approach. Across districts, between 30–80% of households yielded at least one contaminated sample. Aflatoxin B1 detection rates and mean contamination levels were highest in groundnut and maize, respectively, and lower in other crops. Landholding had a positive univariate effect on household aflatoxin detection, while storage conditions, product source, and the number of protective behaviors used by households did not show significant effects. Presence of groundnut, post-harvest grain washing, use of sack-based storage systems, and cultivation status (farming or non-farming) were identified as the most contributive variables in stepwise logistic regression and were used to generate a household-level risk index. The index had moderate classification accuracy (68% sensitivity and 62% specificity) and significantly correlated with village-wise aflatoxin detection rates. Spatial analysis revealed utility of the index for identifying at-risk localities and households. This study identified several key features associated with aflatoxin contamination in Indian food systems and demonstrated that household characteristics are substantially predictive of aflatoxin risk.

## Introduction

Mycotoxins are fungal metabolites that can contaminate a range of food products. Aflatoxins, produced by *Aspergillus flavus* and *A*. *parasiticus*, are a class of potent hepatotoxic mycotoxins that have been implicated in chronic and acute health problems [1]. Aflatoxins can accumulate in foods at any stage along the value chain. Pre-harvest aflatoxin deposition is influenced by crop growing conditions and by the genetics of the host-pathogen interaction [2, 3]. Post-harvest contamination occurs if products are exposed to sub-optimal (particularly moist) conditions during harvest, drying, processing/handling, and storage [4]. Aflatoxins have been documented in food systems spanning the tropics and sub-tropics, including the South Asian sub-continent [5–7]. Several aflatoxicosis outbreaks have occurred in India in the past [8, 9]. Although there is widespread appreciation of aflatoxins as a public health concern and a few intervention studies have shown promising outcomes [10–12], no mitigation strategies have achieved widespread adoption by vulnerable populations [13].

Most intervention efforts are limited to a narrow focus on a single crop or a fixed point along a food value chain. Given the known high susceptibility of some plant hosts, particularly maize and groundnut [14], these have received the bulk of attention and resource allocation for aflatoxin mitigation. However, there is evidence that less susceptible crops such as rice, sorghum, and millets, which are important staple foods in many parts of the world, can contribute substantially to the dietary aflatoxin burden [15, 16]. As mycotoxin contamination within and across food systems is dynamic and influenced by a range of pre-harvest and post-harvest food system features [11], inability to capture risks associated with these features may be a reason that existing interventions have not led to scalable improvements.

India is a large, highly populated country in South Asia with rich cultural and biophysical diversity that is mirrored in its many food systems [17, 18]. In North India’s Indo-Gangetic Plains region, smallholder agricultural economies largely follow the rice-wheat model ushered in by the Green Revolution in the 20^th^ Century, which is prone to low food system diversity [19]. Moving southward, cropping systems are more diverse: rice is ubiquitous, along with pockets of coarse grains, sugarcane, cotton, and other food and cash crops that emerge regionally [20]. The spatial variation in food system composition corresponds to diverse sociocultural practices underpinning food production, preservation, sale, preparation, and consumption. These features potentially influence the nature of aflatoxin exposure in a locally specific manner, necessitating surveillance systems that can inform local intervention actions.

Prediction modeling has emerged as a powerful tool for locally specific aflatoxin risk assessment in a number of contexts. Models have been developed to estimate pre-harvest aflatoxin risk based on environmental data related to weather (drought stress is a major driver of aflatoxin accumulation), land forms (altitude; aspect), soil characteristics, and vegetation cover [21, 22]. A major environmental determinant jointly influenced by agroclimatic forces is the interaction between soil moisture and soil temperature, which have strong relationships with AFB1 accumulation [23, 24]. Moreover, a range of post-harvest risk factors have been identified, although their utility in prediction modeling has not been investigated in depth. Aflatoxin accumulation in grain after harvest is influenced by drying practices, sorting and processing, and by characteristics of the storage environment [4, 25]. Each of these risk factors is potentially variable within and across spatial scales, suggesting that they may be useful for predictive modeling.

A limitation of prediction modeling in aflatoxin risk assessment is that models are not typically developed at scales that enable identification of specific, locally meaningful intervention options. Landscape-scale spatial models constructed from remotely-sensed data do not acknowledge site-specific agronomic management practices [22]. At the opposite extreme, sample- or batch-scale prediction models, often based on spectral signatures [26, 27], are specific to individual crops and are not informed by food system characteristics. Modeling risk at the household level can complement regional models by identifying specific factors that households can control and that can be targeted by intervention programming. A number of community-based interventions focused on such risks have led to successful reductions in aflatoxin exposure in vulnerable communities [12, 28–30], but all methods may not be equally effective across contexts. Risk prediction models based on household characteristics could contribute both to surveillance and to the identification of locally meaningful aflatoxin management strategies for target communities.

To date, no procedure has been developed for assessing aflatoxin contamination risk at the household level. Availability of such a tool to stakeholders engaged in monitoring food safety and community health could support gains in both aflatoxin awareness and in the deployment of behavior change interventions that are responsive to locally specific drivers of contamination. Several established household-level indices for other types of risk have exemplified this potential. The dietary diversity index, for example, is a common score-based household risk indicator that is associated with a range of nutritional and social outcomes in Indian populations [31, 32]. With only a brief household-level interaction, field practitioners can compute dietary diversity scores and evaluate their components, which immediately highlight risk areas and intervention opportunities [33]. Analogous to the relationships between dietary diversity and health outcomes, there are food and crop preservation characteristics that could serve as indicators of household-level vulnerability to aflatoxin exposure.

Spatial analysis of household-level risk factors could complement existing indices of aflatoxin risk developed using landscape- and sample-scale predictors. Integrating household data with data sets across scales, such as remotely-sensed data or census data, has previously enabled investigations of the sociocultural drivers of biophysical phenomena [34, 35]. In the Brazilian Amazon, de Souza Soler and Verberg [36] paired remote sensing data with household-level characteristics to identify relationships between land use history and deforestation. While such integration across scales has proven effective in other contexts, there remains a gap in our understanding of the nature of household-level aflatoxin exposure risk, and the predictive value of household characteristics in risk modeling. An initial step toward integration of the human element of exposure risk into spatial surveillance systems is to characterize household-level risk factors and to determine their relationships with aflatoxin contamination across spatial scales.

In this study, we sought to achieve a comprehensive understanding of the various household-level drivers of aflatoxin contamination across a range of Indian smallholder food systems. We characterized several known risk factors pertaining to food system dynamics (e.g. crop types, storage conditions, sources, etc.) and sociocultural characteristics (e.g. crop protection and food preservation behaviors, socioeconomic status, etc.), and evaluated relationships between these factors and aflatoxin contamination status. We then developed and validated a household-level aflatoxin risk index based on these risk factors, which could help in identifying at-risk households and communities that would benefit from behavior change interventions.

## Methods

### Target areas

Sites for survey implementation were selected based on their distinctiveness from both agroecological and sociocultural standpoints, as well as on the pre-established relationships with local NGOs that were essential for facilitating entry to and mediating interactions with stakeholder communities. Maharajganj District is located in the northern Indian state of Uttar Pradesh, in the fertile Indo-Gangetic Plain region. The region, spread across 2,952 km^2^, has a total population of 2,173,000 [37, 38]. Maharajganj has mean annual rainfall of 850 mm and an average elevation of 96 m above sea level. Rice and wheat are the major commodities in this region both in terms of production and consumption. Munger District is situated along the Ganges River in central Bihar, with a mean annual rainfall of 1,143 mm and average elevation of 45 m above sea level [39]. According to the 2011 census, the district has a total population of 1,359,054. As in Maharajganj, rice and wheat predominate in Munger District, with maize an occasional supplement to wheat flour. Kandhamal District is located in the forested inland region of central Odisha. The population of the district is 731,952 according to the 2011 census. Kandhamal sits at 553 m elevation and receives a mean annual rainfall of 1,727 mm [40]. This district is relatively isolated, and members of “scheduled tribes” (ethnic minorities) account for a significant fraction (54%) of the population [38]. Rice is the major staple grain in the region, whereas wheat is generally neither produced nor consumed. Mahabubnagar District is a large district (18,432 km^2^) in the southern Indian state of Telangana. The district has a total population of 4,053,028 according to the 2011 census. Mahabubnagar has an average elevation of 497 m and a drier climate, receiving just 692 mm rainfall as an annual average [41]. The cropping system of Mahabubnagar is more diverse than the other three districts, with rice, sorghum, and pulses produced as major food crops. In addition, castor, groundnut, and sugarcane are common cash crops in the region.

### Ethics

As the survey objectives were to document only methods procedures associated with food storage (and not the participants’ opinions and decisions, or how the methods affect them or their environment), this study did not qualify as research with human participants according to the guidance of the Cornell University Institutional Review Board and therefore no review was necessary. All survey participants were made aware of the scope and purpose of the study before participating and gave oral consent. Oral consent was documented within the survey questionnaire prior to each interview. Written consent was not sought due to literacy constraints in the population.

### Household selection and survey administration

Household surveys were conducted between June-August, 2016. Nine villages across the four districts (two each in Maharajganj, Munger, and Kandhamal, and three in Mahabubnagar) were identified. Within each district, we aimed to survey 30 households (~15 per village) representative of the range of castes and socioeconomic profiles in each locality. We used a stratified random sampling approach to select household respondents grouped into three socioeconomic strata as determined by the household landholding and head of household’s occupation. The number of households in each stratum was approximately representative of the class composition of each village. Both farming and non-farming households were included in the survey. We also considered the spatial distribution of survey households within the village and selected households such that the village coverage was as comprehensive and uniform as possible. In total, 160 households were recruited for the study, with 39, 39, 31, and 51 households from Maharajganj, Munger, Kandhamal, and Mahabubnagar, respectively.

Prior to survey data collection, each respondent was briefed on the general nature and objective of the survey effort. Interviews and sample collection were conducted on a voluntary and consensual basis. Upon receipt of oral consent, a questionnaire (S1 File) was administered orally in an interview with the head of household or spouse in their native language. The corresponding survey data can be found in the S1 Dataset. Following the interview, respondents were asked to submit 50 g samples of all staple food products present in the household (usually between 2–10 samples per household) for aflatoxin analysis (S2 Dataset). Information regarding the history, consumption, and handling of each sample was collected using a brief questionnaire. Items collected, if available, included rice, wheat, pulses, sorghum/millet, maize, groundnut, sesame, and mustard. Given the diversity in size and type of storage vessels from which samples were drawn, we systematically collected from the first portion to be consumed (i.e. one deep handful from top of sack or from dispensing spout of metal bin, *etc*.). Samples were placed immediately into a sterile plastic sample pouch and stored under refrigeration until analysis. After the interview and sampling process, each respondent was given a steel bowl as compensation for interview participation and the sampled grain.

### Sample processing and aflatoxin B1 analysis

Each sample was ground to fine powder (approximately 820–850 μm, or fine enough to pass through a 20-mesh sieve) using a sterile laboratory blender. Blenders and utensils were sanitized after each sample using 70% ethanol. Ground samples were immediately returned to their original pouches for subsequent mycotoxin extraction. Aflatoxin B1 (AFB1) extraction was conducted using a protocol described by ICRISAT. After grinding, 10 g of each sample was transferred to an Erlenmeyer flask, and mixed with 50 ml of 70% methanol containing 0.5% KCl. Flasks were shaken for 30 minutes at 300 rpm, and the extract filtered through Whatman No. 4 filter paper. Extract filtrates were stored at 4°C prior to analysis.

To quantify AFB1, we used an indirect competitive enzyme-linked immunosorbent assay (ELISA) procedure developed by ICRISAT [42]. AFB1-bovine serum albumen (BSA) was prepared in carbonate buffer at concentration 100 ng/ml, and 150 μl was added to each sample well of ELISA microtiter plates and the plates incubated for 1 hour at 37°C. Phosphate-buffered saline with Tween 20®-BSA (PBST-BSA) was added to each plate and incubated at 37°C for 30 minutes. AFB1 standards (concentrations 25–0.097 ng/ml) were prepared in PBST-BSA with 7% methanol and added to the test plate in 100 μl quantities. Sample extracts were diluted 1:10 in PBST-BSA, and 100 μl was added to the sample wells. The antiserum was diluted 1:6,000 in PBST-BSA, and 50 μl was added to each well. The plates were incubated again for 1 hour at 37°C. 150 μl of the enzyme conjugate anti-rabbit-IgG-ALP (diluted 1:4,000 in PBST-BSA) was added to each well and incubated at 37°C for one hour. Substrate p-nitrophenyl phosphate prepared in 10% diethanolamine was added to the wells, and the plates were incubated for 20 minutes to allow the color reaction to develop.

Absorbance was read at 405 nm using a Bio-Rad iMark microplate reader (Bio-Rad Laboratories, CA, USA). The limit of detection (LOD) for this assay was 0.1 μg/kg and the limit of quantification (LOQ) was 1 μg/kg [43]. Optical Densities (OD) for all samples (in duplicate) were processed using the Microplate Manager 6 software (Bio-Rad Laboratories, CA, USA). Sample concentrations were calculated by interpolating on second-order polynomial standard curves generated for each plate. Samples with OD values outside the OD range of the standards were serially diluted and re-analyzed, and the dilution factors adjusted accordingly in the calculation.

### Identification of aflatoxin risk factors

Several household-level risk factors were identified *a priori* as possible drivers of aflatoxin contamination in local food systems based on evidence from previous studies. We prioritized indicators that could be readily gathered in a brief interview. Because differential crop species susceptibility is known to influence aflatoxin contamination outcomes, we evaluated the distribution of crop species within and across households. The usage of various types of traditional and modern storage facilities was examined, as it has been shown that container types differ in their vulnerability to fungal colonization and aflatoxin accumulation [44–47]. Toxin-producing strains of *A*. *flavus* can proliferate in storage under sub-optimal conditions, and a positive linear relationship between aflatoxin concentration and storage time has been observed [48, 49]. Therefore, storage time in days (averaged over all samples in each household) was also computed for each household.

The source details for all samples were collected during the interview process, and categorized into five groups: homegrown, market, gift (from friends and family), public distribution system (PDS), and as wages. For household-level analyses, we used the proportion of homegrown samples as an indicator of risk associated with grain source. We used landholding (hectares) as a proxy variable for household socioeconomic status, as it is a reliable indicator of stable household wealth in India [50]. We hypothesized that households deploying more crop protection and food preservation behaviors would be less likely to have detectable aflatoxin in their grain stores, and therefore counts of unique crop protection and food preservation behaviors were taken in each household as indicators of agronomy- and food safety-related risk levels, respectively.

### Statistical analysis of aflatoxin risk factors

For the crop-level risk factors (crop species, storage container, storage time quantiles, and grain source), analysis of variance (ANOVA) was used to determine whether there were significant differences. To control for crop-wise effects in the ANOVA models (except for the crop species ANOVA), crop species was included as a blocking/nuisance factor to minimize the variability conferred to the response variable (aflatoxin concentration). For all quantitative analysis, aflatoxin concentrations were transformed by log_10_(x+1) to normalize the distribution of observations, as has been described previously [51]. ANOVAs were performed in the R software environment using the car package [52]. Multi-level logistic regression models were constructed for storage container, storage time, and grain source to test for fixed effects on the odds of sample AFB1 detection status (≥ 1 μg/kg). Crop species and sampling location were included as random effects to account for non-independence of observations. Modeling was completed using the glmer function in the lme4 R package [53].

A similar multi-level modeling approach was used to test for significant univariate effects of the household-level risk factors (landholding, crop protection practices, and food safety practices) on AFB1 detection in the household. Household values were used as fixed effects, with household AFB1 detection status (i.e. whether at least one sample was contaminated in the household) as a binary dependent variable. District was included as a random effect to control for possible similarities among households surveyed in the same locality. Models were fitted in R as described above. A threshold value of p ≤ 0.05 was used to signify statistical significance of all tests.

### Index selection and validation

Our aim was to develop and validate an index for predicting whether at least one sample collected in a household was contaminated (≥ 1 μg/kg) with AFB1. A total of 28 variables were considered for prediction modeling based on the *a priori* risk factors described. Categorical/binary variables with <5% coverage in the household data were omitted. Binary variables for presence/absence of crop species in the household (wheat, maize, groundnut, sorghum, and pulses) were included as indicators of species vulnerability. Rice was omitted because only 2% of households contained no rice. The presence/absence of crop protection behaviors (fertilizer use, pesticide use, and good agronomic practices) and food preservation behaviors (sorting, drying, washing, clean vessels, chemical additives, and natural additives) were included as indicators of behavioral risk. Usage of certain storage containers (sacks, boxes, traditional, and other modern) were also included as binary variables. Household cultivation status (farming/non-farming) was included as a binary variable. Average household storage time, the proportion of home-grown produce in the household, the number of hectares of land, the number of household residents, the number of months of food insufficiency, and the number of months of food quality inadequacy were included as household-level numeric variables.

Stepwise logistic regression models of household AFB1 detection (Y/N) were constructed using the stepAIC function in the MASS R package [54]. The final model of most contributive variables was selected based on Akaike information criterion (AIC). Variables selected in stepwise regression that reached a significance level of p < 0.05 were taken forward for risk index development. Risk index values were developed for each selected indicator by taking the square root of the odds ratio estimated in the reduced model, as previously described [55]. Household risk scores were computed by summing the index values.

A repeated 5-fold cross-validation approach was used to evaluate the performance of the composite and disaggregated indices, adapted from a method described previously [56]. *K*-fold cross-validation is a useful strategy for evaluating predictive performance in small datasets and has better variance and bias properties than alternatives such as leave-one-out cross-validation [57]. The data were split into *k* = 5 groups, and each group iteratively used as a validation set for models fit on the remaining *k*-1 = 4 groups. This procedure was repeated 100 times, re-shuffling the observations each time, in order to obtain reliable prediction estimates [58]. We used area under the receiver operating characteristic (AUROC) curve as a measure of model accuracy. Sensitivity (households with truly detectable AFB1) and specificity (households with truly no detectable AFB1) were computed at the point on the ROC curve where both were maximized for each cross-validation fold and iteration. Performance thresholds of 60%, 80%, and 90% for these indicators were taken to represent moderate, good, and excellent classification accuracy, respectively. Model training, predictions, and classification accuracy evaluation were performed using the caret package in R [59]. Additionally, a Pearson correlation test was performed to evaluate the relationship between index scores and observed local household AFB1 detection rates.

As our aim was to validate a risk indicator that is readily interpretable to users without statistical modelling expertise, we chose to pursue a composite risk index score constructed by summing all index values. If validated properly, summative scores can be a convenient and accurate way to predict risk levels [60]. However, we acknowledge that summative indices can sometimes have reduced predictive power compared to modeling risk factors as individual model covariates. To determine whether and to what extent prediction accuracy was reduced in the composite score compared to the selected indices modeled independently, we compared classification performance results of the composite index score, described above, to those of a prediction model fitted with the selected indices as disaggregated covariates.

### Spatial risk analysis

Global positioning satellite (GPS) coordinates were recorded for each household at the time of sampling using a handheld GPS system. Risk index scores were computed for all households as described above. Observed household detection status and index prediction accuracy were mapped and compared to visualize regional performance of the index. District-level household AFB1 detection rated and mean household risk index values were calculated and visualized using the ggmap package in R [61]. Intra-village risk distribution was evaluated by mapping the spread of index scores within each village. Spatial autocorrelation of household index scores was evaluated within each village by computing Moran’s *I* with the ape package in R [62]. All map vector and raster data used were in the public domain accessed via the rnaturalearth package in R [63] or with permission from the Global Administrative Areas (GADM) database (www.gadm.org). Differences in mean index scores among districts were assessed using ANOVA and post-hoc Tukey tests for multiple pairwise comparisons. ANOVA (essentially a t-test when only two groups are being compared) was used to compare village-wise mean index scores within districts.

## Results

### Aflatoxin contamination

**Fig 1:**
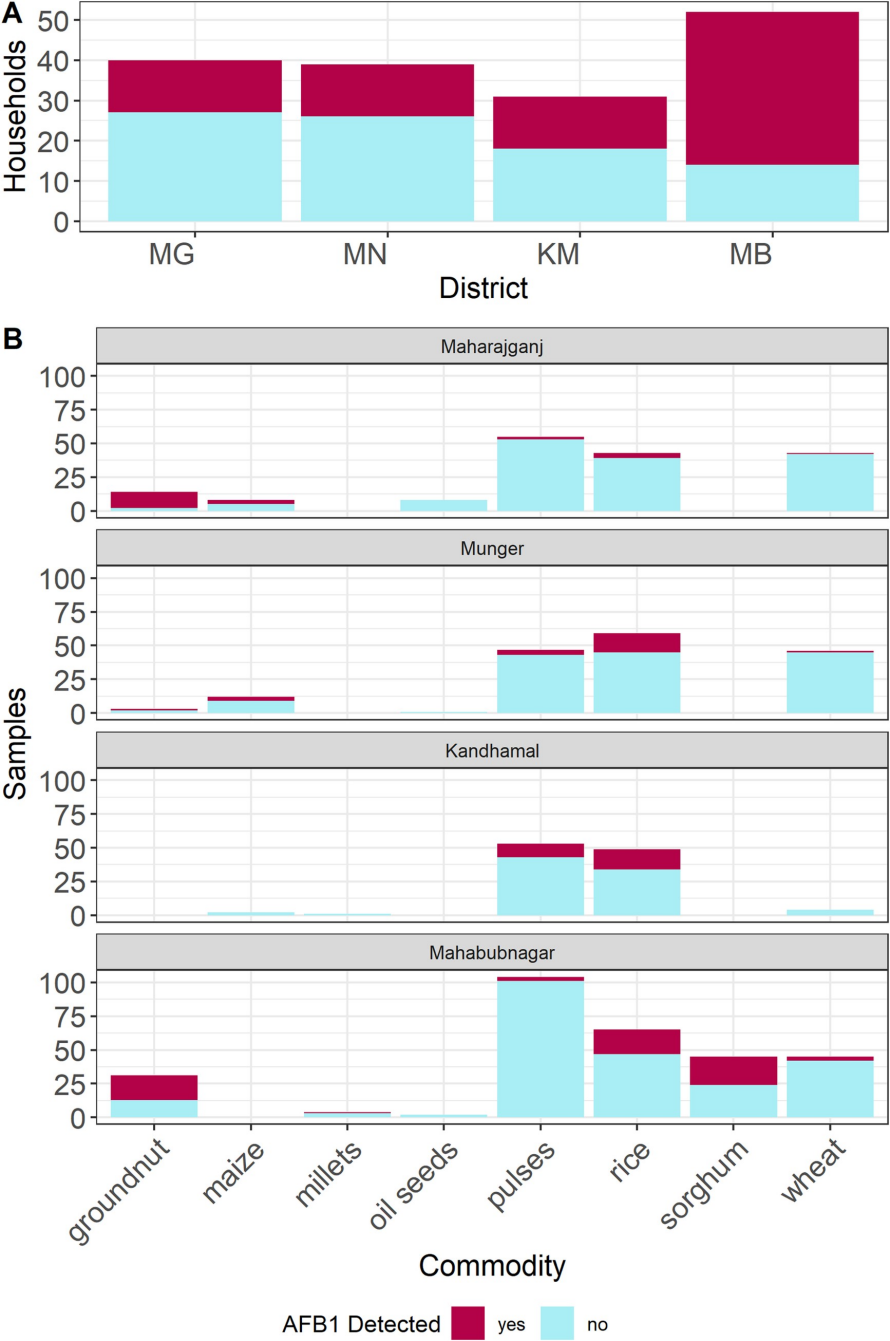
Household- and sample-wise aflatoxin detection rates across localities. (A) Proportion of households that yielded at least one sample with detectable AFB1 (>1 μg/kg). MG = Maharajganj, MN = Munger, KM = Kandhamal, and MB = Mahabubnagar. (B) Detection by district of AFB1 across samples in eight major food crops.

To increase our understanding of the diversity of Indian food systems, we surveyed the foods present in 160 households in 9 villages across 4 districts in summer 2016. Among the 595 samples obtained, the most commonly available food items were rice, pulses, and wheat, constituting 190, 174, and 128 samples, respectively. Sorghum (n = 38), groundnut (n = 31), maize (n = 17), oil seeds (e.g. mustard or sesame); n = 13), and pearl millet (n = 4) were present in far fewer households. In each district, more than 30% of households yielded at least one sample with detectable (>1 μg/kg) aflatoxin B1 (AFB1) levels (Fig 1A). Mahabubnagar had the highest incidence of household-level mycotoxin detection, with contaminated samples collected from 82% of households. This high rate reflected the relative abundance of groundnut and sorghum (which are highly susceptible to toxin accumulation) in this region. The other three districts had much lower but still substantial rates of household-level aflatoxin detection (< 50% of households). The commodities typically associated with aflatoxin accumulation, such as groundnut, maize, and sorghum, had high incidence of contamination across study sites (Fig 1B). While we observed some contamination in rice and pulses samples, wheat was contaminated at very low frequency and was therefore an unlikely source of dietary aflatoxin under normal conditions. There was low prevalence (9%) of households yielding samples contaminated above the regulatory legal limit (15 μg/kg), and therefore we did not have enough observations to validate predictive models for legal/illegal regulatory status.

**Fig 2:**
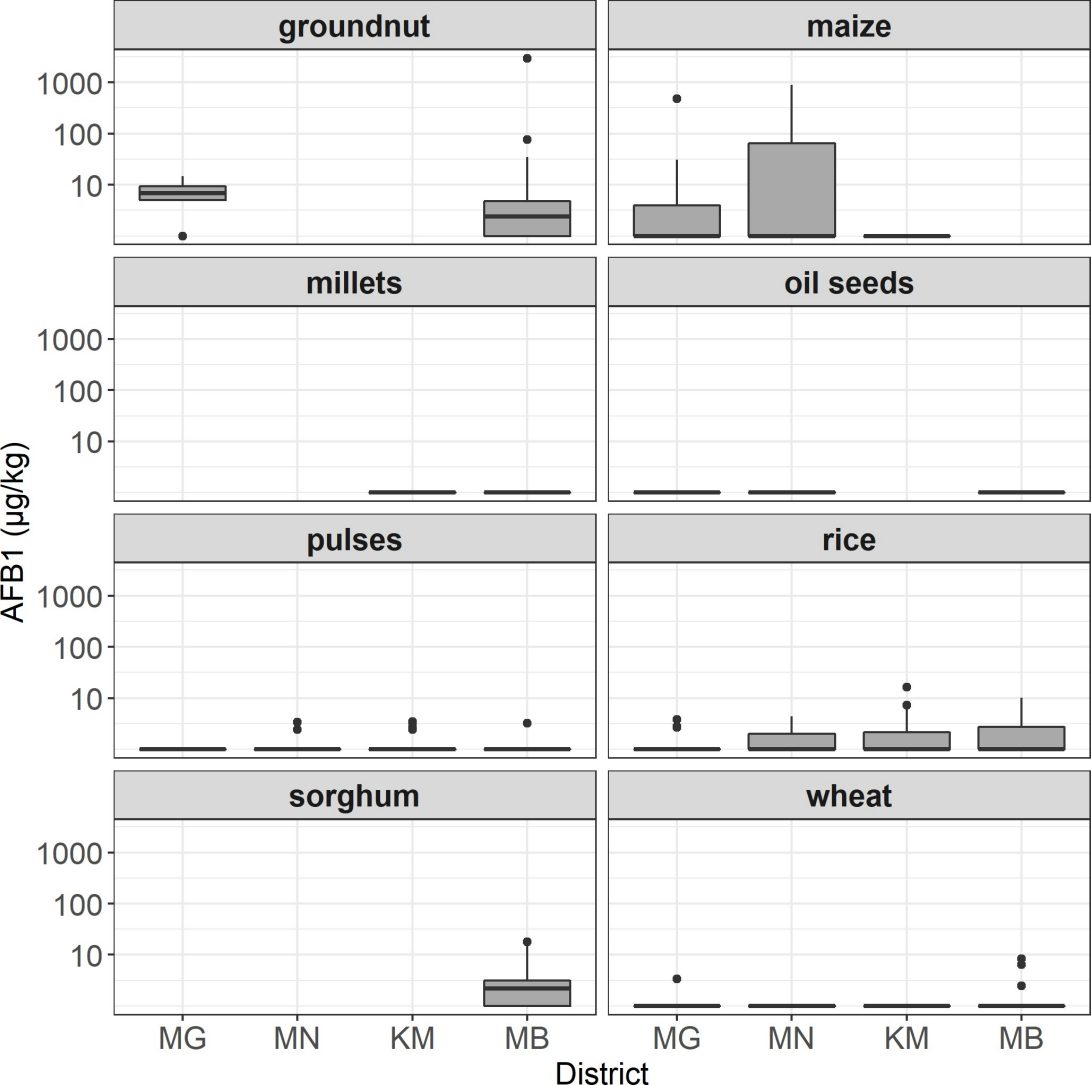
Range of AFB1 contamination across districts by crop group. District codes MG = Maharajganj, MN = Munger, KM = Kandhamal, and MB = Mahabubnagar.

There appeared to be some regional trends in vulnerability of grain groups to aflatoxin accumulation (Fig 2). For example, groundnut and maize samples were much more highly contaminated in Munger than in Mahabubnagar, perhaps owing to the more humid storage conditions in Munger or to the relatively low importance of this crop in the Munger food system compared to Mahabubnagar. Rice and pulses had the highest detection rates and contamination levels in Kandhamal, where these crops were the predominant staple foods.

The magnitude of aflatoxin contamination in all commodities was generally below the Indian regulated legal limit of 15 μg/kg, but 18% of samples exceeded this limit. The most heavily contaminated samples yielded AFB1 concentrations approaching 3,000 μg/kg. As expected, groundnut and maize were the most severely contaminated commodities, comprising most of the samples that were contaminated at levels exceeding the regulatory limit. Sorghum and rice were frequently contaminated in the 5–10 μg/kg range, suggesting that these commodities may be moderate contributors of dietary aflatoxin in local food systems. While some commodities were more prevalent in some districts than others, we did not detect significant differences in crop-wise AFB1 contamination levels among districts where a given crop was present (p > 0.1). This suggests that presence/absence of susceptible crops in a food system is more important than environmental effects on specific crops in determining a community’s risk profile.

### Household dynamics and food systems

Surveyed households represented a range of socioeconomic strata and living arrangements within and across districts. The average head of household age was 47, and there were no statistically significant differences across districts with the exception of Maharajganj (mean age 56, p < 0.05 in pairwise comparisons). Multi-generational, joint, and nuclear households were the most common household types among survey respondents, accounting for 88–100% of all households in the sample across districts. Other household types were less common, including elderly couples, single individuals, and single parents with children. Households in the sample had 6.3 members on average, with no significant differences between districts.

Across all sites, 41% of households reported having insufficient food to meet the family’s needs at least one month per year. In Mahabubnagar, where many of the study households predominantly relied on cash crops for supplemental income, 70% of households reported food insufficiency. In contrast, Maharajganj had no households reporting insufficient quantity of food in any month(s). Munger and Kandhamal fell in the middle, both with 42% of households reporting food insufficiency. On average, the households surveyed in this study reported food shortage in one (of possible 12) month of the year. The majority of households reporting food quantity insufficiency indicated that the highest risk is during the summer months (March—June). There was lower regional variation in perceived food quality insufficiency, recorded as the number of months in which perceived food quality (i.e. safety and nutritional value) was sub-optimal. Maharajganj yielded the lowest proportion of households that perceived low food quality in at least one month of the year (24%). The remaining districts Munger, Mahabubnagar, and Kandhamal had values of 34, 38, and 39%, respectively. Munger had marginally higher mean number of “low-quality months” than the other study sites (p < 0.1 for all pairwise comparisons), with a mean of 3.3 months compared to 0.9, 1.3, and 1.5 months in Mahabubnagar, Kandhamal, and Maharajganj, respectively. Similarly to food quantity insufficiency, food quality issues were reportedly most prevalent during the summer months.

**Fig 3:**
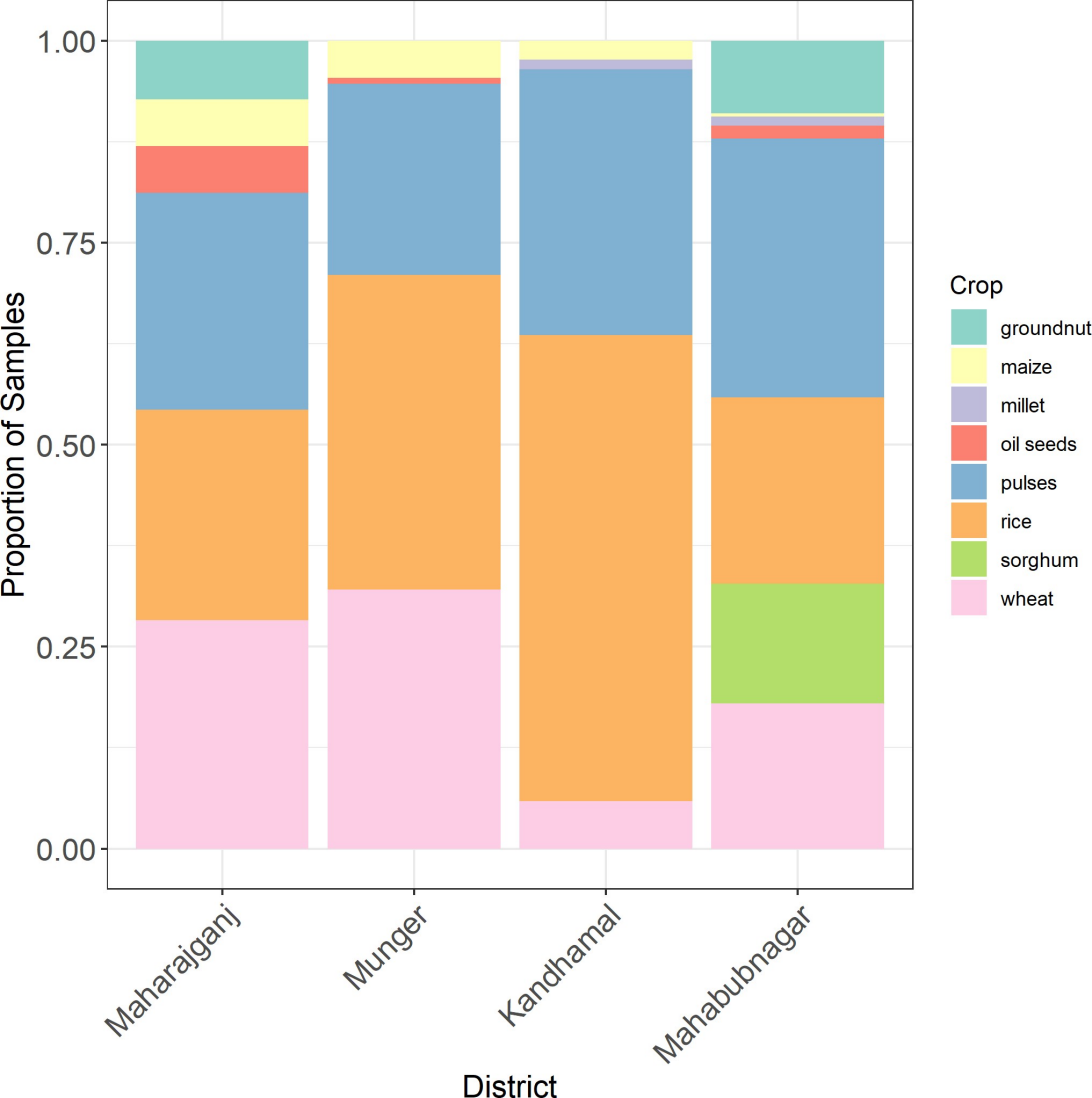
Regional distribution of sample yield across grain groups. Percentages represent the fraction of household-derived samples collected in respective districts.

As the samples collected in each household generally reflected all staple food commodities present in the household, sample yield was used as an indicator of regional household consumption characteristics. Consumption of rice and pulses was relatively uniform across sampling sites (Fig 3). Consumption of wheat and sorghum, the other major staple food commodities, was more region-specific. Respondents from Kandhamal provided very little wheat, indicative of a strong reliance on other staple commodities (especially rice) for dietary energy. Sorghum was exclusively present in Mahabubnagar households, reflecting the drier growing conditions and cultural preference for coarse grains over wheat in this area. Among non-staple food commodities, maize and groundnut were the most common. Maize was not widely cultivated or consumed in any region but was present for human and livestock consumption in low quantities across all study sites. Respondents reported that maize consumption was markedly seasonal, and generally consumed in the form of mixed maize/wheat or maize/sorghum bread (*roti*). Groundnut was a very popular food among Mahabubnagar respondents, as the district was a major regional producer of this crop. Groundnut was also widely cultivated and consumed as a non-staple food item in Maharajganj.

### Crop production systems

Households were considered to be self-provisioning if at least one staple grain item (rice and/or wheat) was produced on the farm and consumed by the household. Across all districts, at least half of households included in the sample reported some degree of self-provisioning. This was least common among households in Mahabubnagar District (54%), where cash crop production was a major source of income and many households were reliant on local markets and the Public Distribution System (PDS) for staple food items. Munger and Kandhamal had stronger majorities of self-provisioning households, accounting for 63% and 65% of households, respectively. Self-provisioning was most common in Maharajganj, where 92% of households reported producing staple grains on their own farm for household consumption.

Crop production for all districts is summarized in Table 1. Rice was the most widely cultivated *kharif* (rainy) season crop across all districts, grown by 68–95% of surveyed households. Other common *kharif* crops included pulses, maize, groundnut, and vegetables. Crops grown in the *rabi* (post-rainy) season were more diverse across regions, reflecting cultural preferences and environmental constraints. Wheat was commonly cultivated in the *rabi* season in the northern regions (Maharajganj and Munger) but was far less frequent in Mahabubnagar and Kandhamal. In Kandhamal, rice was strongly preferred over wheat-based *roti* as a staple food, and respondents often reported that wheat was prohibitively expensive in the marketplace. Respondents in Mahabubnagar preferred rice or sorghum-based *roti* over the wheat-based alternative. Potato was another commonly cultivated *rabi* season crop in all districts except Mahabubnagar, where it was generally avoided and often considered unhealthy. During the summer season, few households in our sample reported cultivating any crops. Among the households that did cultivate during the summer season, vegetables, maize, and potato were the most popular. Notably, no cultivation during the summer season was reported among Mahabubnagar households, owing to unfavorably hot and dry conditions during the summer months.

**Table 1:** Crop production across cropping seasons for each study location.

District	Total HH	*Rainy*	*Post-Rainy*	*Summer*
Maharajganj	38	Rice (94.7%) Groundnut (65.8%) Pulses (52.6%) Maize (42.1%) Vegetables (34.2) Sorghum (2.6%)	Wheat (94.7%) Vegetables (78.9%) Potato (52.6%) Pulses (23.7%) Mustard (18.4%) Spices (7.9%) Chilies (2.6%)	Vegetables (39.5%) Maize (5.3%) Potato (2.6%)
Munger	38	Rice (73%) Maize (60.5%) Vegetables (50%) Pulses (21.1%)	Vegetables (92.1%) Pulses (65.8%) Potato (63.2%) Wheat (60.5%) Mustard (21.1%)	Vegetables (26.3%)
Kandhamal	31	Rice (67.7%) Pulses (35.5%) Vegetables (35.5%) Maize (19.4%) Groundnut (3.2%)	Potato (35.5%) Pulses (19.4%) Vegetables (16.1%) Millet (3.2%)	Vegetables (3.2%)
Mahabubnagar	50	Rice (68%) Pulses (58%) Castor (30%) Chilies (8%) Sorghum (4%) Sugarcane (2%) Cotton (2%) Vegetables (2%)	Rice (44%) Groundnut (26%) Vegetables (6%) Castor (2%)	

Numbers in parentheses indicate percentages of surveyed households (HH) in each district that reported growing the crop in the specified season.

### Food storage practices

Because storage conditions are known to be an important risk factor associated with post-harvest mycotoxin accumulation, we sought to understand how common food commodities were stored and handled across the study sites. While storage practices varied substantially across geographical regions and crop groups, some commonalities emerged among the households included in the present survey. Table 2 summarizes grain storage systems for all samples collected in the survey. In Munger and Maharajganj, closed tin or plastic containers, or “boxes,” were the most popular vessels for storing staple grains (rice and wheat). In Mahabubnagar, rice and sorghum were most commonly stored in sacks, probably because of the larger quantities procured by householders in this region. Wheat in Mahabubnagar was typically acquired in relatively small quantities via the public distribution system (PDS), which provided grain at subsidized rates; thus, most households kept wheat stored in tin or plastic containers. In Kandhamal, the observed storage conditions for staple grains were less uniform: the fractions of rice and wheat samples stored in sacks and tin/plastic containers were similar. In every district, a substantial majority of pulse samples (65–84%) were kept in tin or plastic boxes. Commodities kept in smaller quantities for occasional use (i.e. groundnuts, millets, oil seeds, etc.) were commonly kept in tin or plastic boxes.

**Table 2:** Summary of grain storage practices across districts.

District	Crop	Storage System
Kandhamal	Maize	Hanging (1/2; 50%), Box (1/2; 50%)
	Millet	Traditional mud (1/1; 100%)
	Pulses	Box (22/28; 79%), Package (3/28; 11%), Traditional mud (3/28; 11%)
	Rice	Box (20/49; 41%), Sack (18/49; 37%), Traditional mud (7/49; 14%), Package (2/49; 4%), Traditional dung (2/49; 4%)
	Wheat	Sack (2/4; 50%), Box (2/4; 50%)
Mahabubnagar	Groundnut	Box (12/23; 52%), Sack (9/23; 39%), Package (1/23; 4%), Traditional pot (1/23; 4%)
	Maize	Pile (1/1; 100%)
	Millet	Box (2/3; 67%), Package (1/3; 33%)
	Oil Seeds	Box (4/4; 100%)
	Pulses	Box (52/80; 65%), Sack (20/80; 25%), Package (8/80; 10%)
	Rice	Sack (50/58; 86%), Box (5/58; 9%), Traditional pot (2/58; 3%), Package (1/58; 2%)
	Sorghum	Sack (29/36; 81%), Box (7/36; 19%)
	Wheat	Box (24/42; 57%), Sack (12/42; 29%), Package (6/42; 14%)
Maharajganj	Groundnut	Box (5/10; 50%), Sack (3/10; 30%), Package (1/10; 10%), Pile (1/10; 10%)
	Maize	Hanging (2/8; 25%), Package (2/8; 25%), Box (2/8; 25%), Pile (1/8; 13%), Sack (1/8; 13%),
	Oil Seeds	Box (4/8; 50%), Sack (3/8; 38%), Basket (1/8; 13%)
	Pulses	Box (30/36; 83%), Sack (6/36; 17%)
	Rice	Box (30/37; 81%), Sack (7/37; 19%),
	Wheat	Box (34/39; 87%), Sack (3/39; 8%), Pile (1/39; 3%), Under Fodder (1/39; 3%)
Munger	Maize	Sack (3/6; 50%), Traditional mud (3/6; 50%)
	Oil Seeds	Box (1/1; 100%)
	Pulses	Box (26/31; 84%), Package (3/31; 10%), Sack (2/31; 6%),
	Rice	Box (23/51; 45%), Traditional mud (15/51; 29%), Sack (12/51; 24%), Silo (1/51; 2%)
	Wheat	Box (16/41; 39%), Traditional mud (13/41; 32%), Sack (12/41; 29%)

In parentheses is the fraction of samples stored using each method, followed by the percent of all samples of the given crop in that district. “Box” signifies a closed metal or plastic container smaller than 100 kg capacity. “Sack” includes 20–60 kg capacity grain storage sacks, typically jute or polypropylene. “Package” signifies a grain stored in a temporary or disposable sealable container, usually in the form of packaged food purchased in the market.

Traditional methods for storing food commodities were practiced in all study sites. Such methods included mud-plastered bamboo silos, mud/dung-plastered bamboo silos, clay pots, and storage under cover of silage/fodder. The mud- and/or dung-plastered structures were particularly popular for storing rice, wheat, and maize. Clay pots were used to store groundnuts and rice. The “under fodder” method was largely used to store wheat in the Maharajganj location.

Using grain type as a blocking factor in ANOVA, we did not observe any difference in aflatoxin contamination levels among storage container types (p = 0.26). There was also no significant effect of container type on the odds of aflatoxin detection in logistic regression (p = 0.33). Significant differences in AFB1 levels across storage time quantiles were observed in maize and rice (p < 0.05). The highest levels of contamination occurred between quartiles 3 and 4 (250–300 days post-harvest) and subsequently decreased. This suggests that there may be threshold value of storage time beyond which contamination levels start to decrease, presumably due to usage or removal of contaminated produce. A similar trend was observed in western Kenya, where the likelihood of aflatoxin contamination in maize was significantly greater at two months post-harvest, but no significant difference was detected after four months [64].

### Grain sources

**Fig 4:**
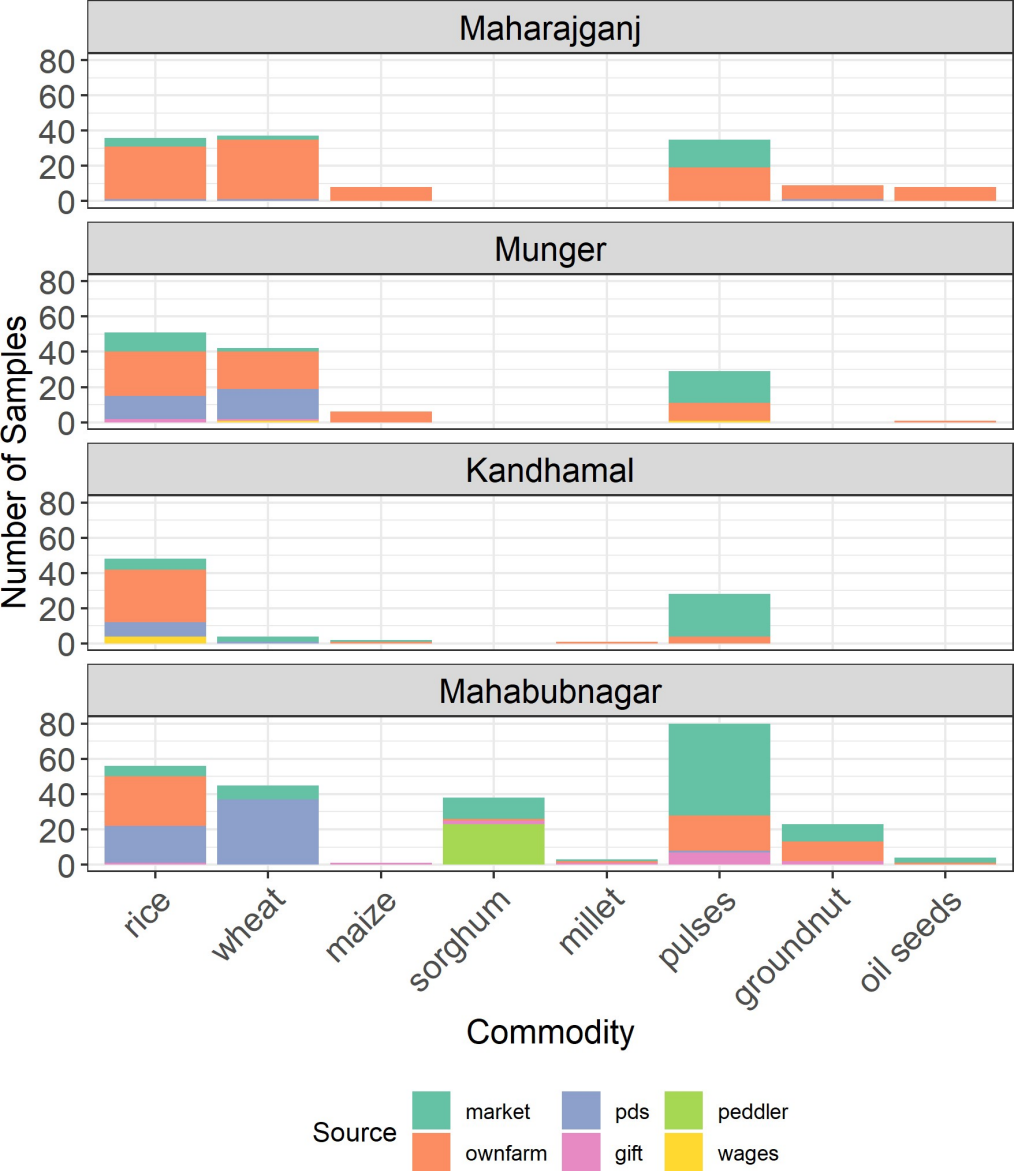
Sources of major food grains. Overview of locality- and crop-wise trends in procurement of food items.

In order to link mycotoxin risk with particular nodes of the food value chain for the commodities of interest in this study, we collected detailed information about the sources of food items in the respective village food systems (Fig 4). In Maharajganj and Munger, where self-provisioning agricultural production was widely practiced, a sizable majority of food commodities was produced on the respondents’ own farms. In contrast, marketplace-derived samples were nearly equal in abundance to those produced on respondents’ farms in Kandhamal. This region, while still predominantly engaged in subsistence farming, was likely more reliant on marketplace-derived food items than Maharajganj and Munger as a result of lower agricultural productivity.

Mahabubnagar households were most reliant on the marketplace for food commodities, followed distantly by on-farm production. This is illustrative of the predominantly cash crop-based smallholder economy in this region. Notably, respondents in Munger and Mahabubnagar Districts were nearly equally reliant on PDS and own-farm production as a source of rice, and Mahabubnagar respondents sourced their wheat products almost exclusively from PDS. A common theme across all study sites was strong reliance on the marketplace for pulses, as opposed to own-farm production or other sources. Interestingly, in Mahabubnagar (the only district where sorghum was consumed), all three villages surveyed in this study relied heavily on traveling peddlers for their sorghum grain, and only a few households reported producing sorghum on their own farms. Among landless/labor class respondents, rice was commonly received as wages for agricultural labor.

Based on ANOVA, there were no significant differences in aflatoxin levels among source categories (p > 0.1). Similarly, there was no detectable difference in the odds of aflatoxin detection between homegrown versus externally acquired samples, controlling for crop type as a random effect (p = 0.94). These findings suggest that contamination levels are consistent regardless of where a household sources its food grains.

### Landholding status

Landholding (ha) was used as a proxy for socioeconomic status. Mahabubnagar had the highest mean household landholding (4.7 ha), corresponding to the higher development status in this region relative to the other districts. Munger had the lowest mean landholding, with only 0.9 ha per household on average. Households in Kandhamal and Maharajganj had average landholdings of 1.5 and 1.6 ha, respectively. The village economies at all sites except those in Mahabubnagar were predominantly based on small-scale farming with high levels of self-provisioning. Cash crop production was the major source of household income in Mahabubnagar. The landless (households with 0 ha of cultivable land) accounted for 11, 12, 34, and 36% of survey households in Maharajganj, Mahabubnagar, Munger, and Kandhamal, respectively. In univariate analysis, landholding had a significant positive effect on the odds of AFB1 being detected in the household (OR 1.35, p < 0.01), suggesting that households with larger landholdings are at greater risk of exposure in these food systems.

### Food and crop preservation practices

**Fig 5:**
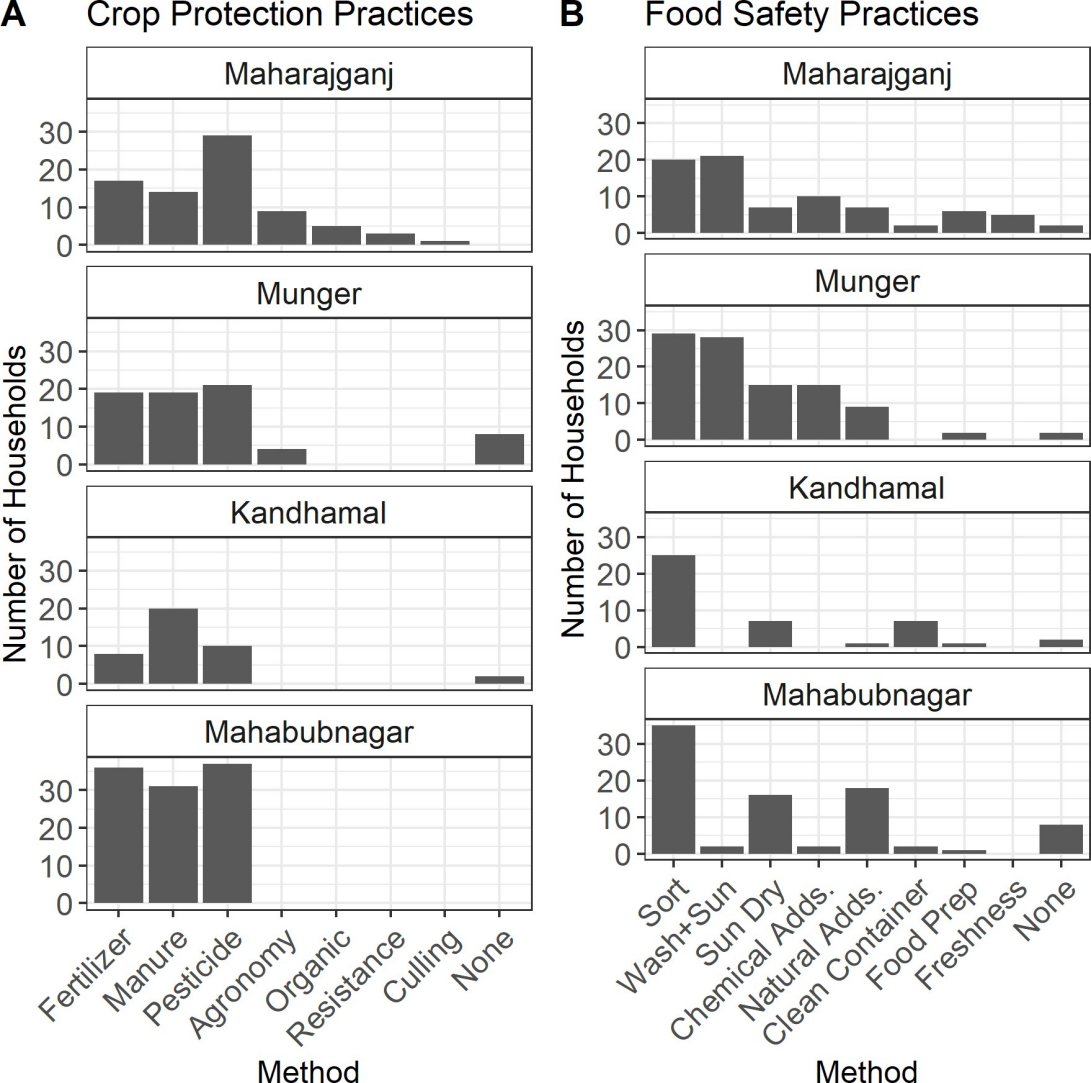
Local food and crop preservation behaviors. District-wise summary of (A) household crop protection practices and (B) household food preservation practices as reported by survey respondents (n = 160).

Diverse crop protection and food preservation practices were reported by households within and across districts (Fig 5). There were, however, no significant effects of the number of crop protection (OR 1.08, p = 0.67) or food preservation practices (1.01, p = 0.96) on the odds of AFB1 detection in the household. The most frequently practiced crop protection strategies were pesticide application (63% of households), manure application (55%), and chemical fertilizers (52%). Nearly all households were using at least one of these methods. Good agronomy was reported as a crop protection strategy by some (<30%) households in Maharajganj and Munger. Maharajganj farmers had the most diverse crop protection practices overall, with some households reporting the use of organic agriculture techniques, resistant varieties, and culling of diseased plants. There were some farming households in Munger and Kandhamal (6% of total households) that did not knowingly practice any crop protection practices despite engaging in crop cultivation.

A similarly wide array of food preservation techniques was being used to ensure household food safety. The most common practices included sorting by hand (70%), washing and then sun-drying (33%), and sun-drying without washing (29%). Farmers across all districts used natural (22%) and/or chemical (17%) preservatives in stored grain. Common natural preservatives and/or insect repellants included neem leaves, ash, and salt. Respondents cited *sulphas* (a popular local term for the fumigant aluminum phosphide; [65]) as a common chemical additive. Compared to the other districts, a relatively high proportion of respondents in Kandhamal cited the use of clean storage containers. Safe food preparation and checking food freshness were commonly reported in Maharajganj, but largely absent in the other districts. Overall, 10% of households did not knowingly practice any food preservation behaviors.

### Household AFB1 detection risk index

#### Prediction model selection and risk index determination

Forward stepwise logistic regression was used to select risk components that were most important for determining household AFB1 detection status. The model selection procedure identified a reduced model consisting of seven risk factors, of which five (presence of groundnut in the household, post-harvest washing, sack-based storage, fertilizer application, and farming/non-farming status) met the p < 0.05 criteria for inclusion in the final prediction model. Because we sought to develop an index that could be applied to both farming and non-farming households, fertilizer usage was discarded and the remaining four indicators were taken forward for risk index development. Presence of maize in the household did not meet p-value inclusion level (p = 0.12), likely due to its relative rareness in the food system and its frequent co-occurrence with groundnut, a highly contributive risk factor. Index values were assigned to each indicator based on the square root of the odds ratio in the model. The scoring system is summarized in Table 3. The final composite index was on a 0–9 scale and was computed for each household. In a univariate logistic regression, we observed a highly significant positive association between the composite index score and the likelihood of aflatoxin detection (OR 1.6, p < 0.0001).

**Table 3:** Odds ratio-based index scoring system for selected risk factors.

Risk Factor	Odds Ratio	Response	Index Value^§^
Groundnut presence in household	7.6	Yes	3
		No	0
Post-harvest grain washing	2.7	Yes	0
		No	2
Use of sack-based storage	2.3	Yes	2
		No	0
Engagement in farming	4.7	Yes	0
		No	2

§ Index values computed by taking the square root of the odds ratio and rounding to the nearest integer.

#### Index validation and performance

A repeated 5-fold cross-validation approach was used to evaluate the performance of the composite and disaggregated household risk indices in predicting household-level aflatoxin detection. The data was split into *k* = 5 random groups 100 times, and each group used as a test set for determining the classification accuracy of a prediction model trained on households in the remaining *k*-1 groups. Both the composite and disaggregated index models classified household AFB1 detection status more accurately than random chance (p < 0.001). Area under the receiver operating characteristic (AUROC) curve was used as a measure of model accuracy. The composite index and disaggregated index models yielded AUROC values of 0.70 and 0.72, respectively. Predictions of household AFB1 detection status based on the composite index score had 68% sensitivity and 62% specificity, indicating respectively that the composite score classified true positives (households with detectable AFB1) and true negatives (households with no detectable AFB1) with moderate accuracy. Sensitivity was similar in the disaggregated model (68%), but specificity was slightly better (64%). There was a highly significant positive correlation (R = 0.94; p < 0.001) between village-level mean risk index scores and observed household AFB1 detection rates, suggesting that the index was a good indicator of local aflatoxin contamination prevalence.

### Spatial analysis of AFB1 risk status

**Fig 6:**
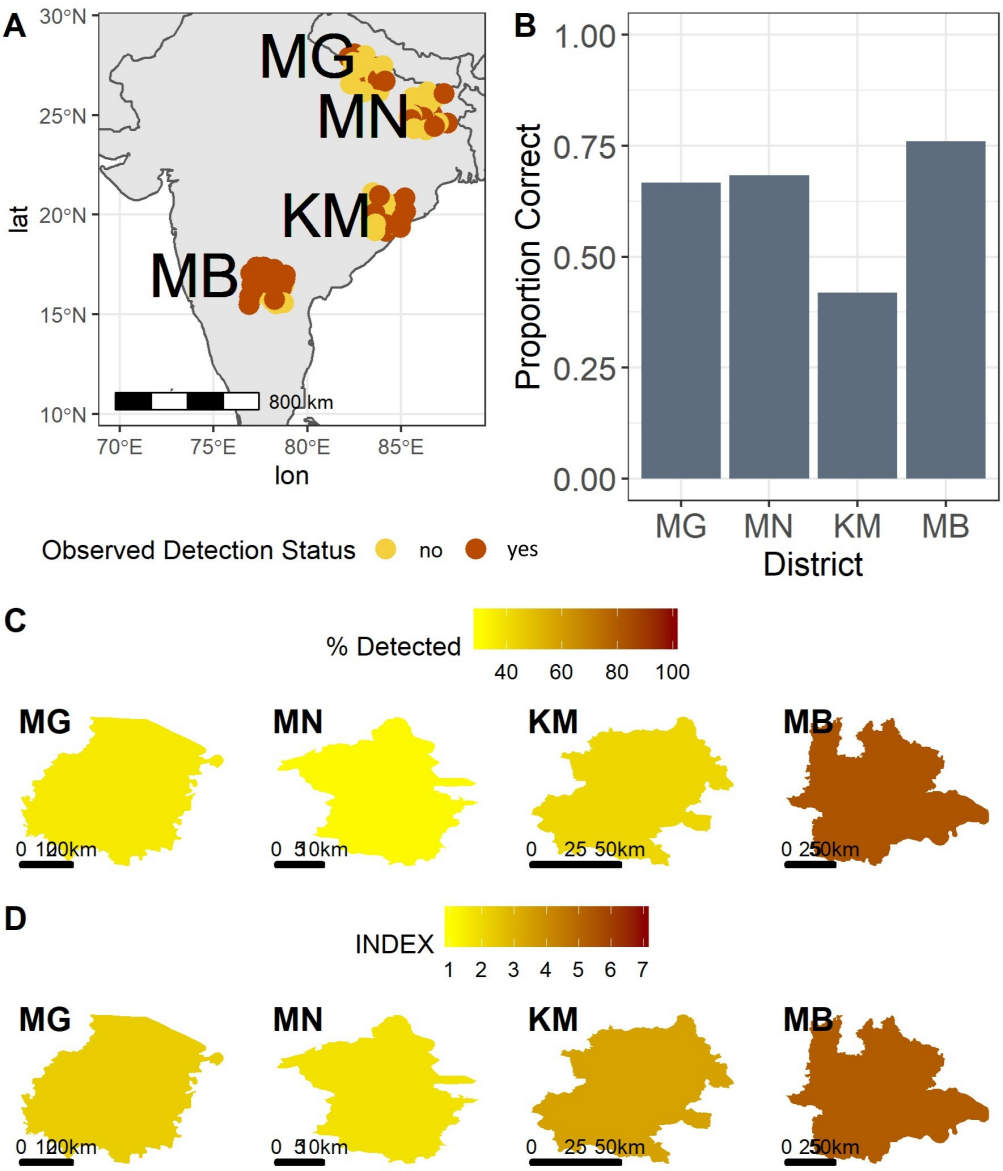
Spatial analysis of household AFB1 detection and risk index scores. (A) Household detection status across localities. Yellow and red points represent households with no detectable AFB1 and detectable AFB1, respectively. Points were jittered to minimize overplotting. (B) Rates of correct household AFB1 status prediction using the risk index, by district. Heat-map representation of (C) district-wise average percent of households with detectable AFB1 and (D) district-wise mean risk index scores. Panel (A) was created using a shapefile from the public domain Natural Earth database (naturalearthdata.com). Panels (C) and (D) use shapefiles reprinted from the GADM database (www.gadm.org) under a CC BY license, with permission from Global Administrative Areas, original copyright 2018.

Overall, there were marked spatial trends in household AFB1 detection status across surveyed districts (Fig 6A), influenced greatly by food system composition as described above. District-level means in composite household aflatoxin risk index values ranged from 1.7 (Munger) to 5.2 (Mahabubnagar), with nearly the full range of possible values (0–8) represented in the population of households. Predictive performance of the risk index was variable by region; the lowest and highest proportions of correctly classified households were observed in Kandhamal (42%) and Mahabubnagar (76%), respectively (Fig 6B). The risk index value can be used to visualize risk levels by district (Fig 6D) and closely approximated observed district-level household detection rates (Fig 6C). All pairwise district index score comparisons were statistically significant (p < 0.05) in *post hoc* Tukey analysis except for Maharajganj and Kandhamal and Maharajganj and Munger.

**Fig 7:**
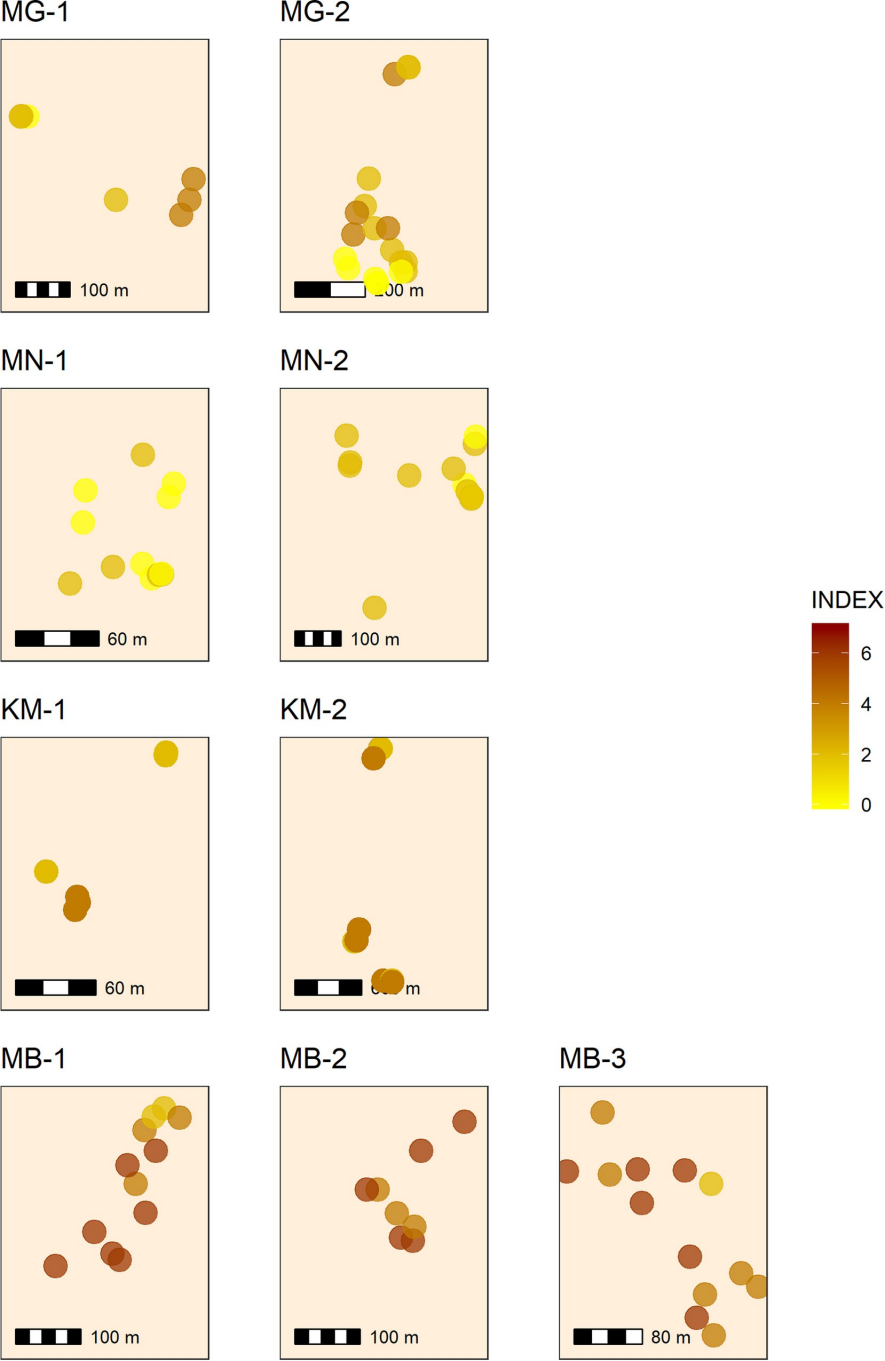
Intra-village distributions of household risk scores. Maps of households sampled in each of the nine villages, indicating the households’ aflatoxin risk index values. District codes MR = Maharajganj, MN = Munger, KM = Kandhamal, and MB = Mahabubnagar.

We sought to determine whether and to what extent risk profiles varied within individual communities, and whether there were spatial trends in risk distribution among households at the village scale. There was significant (p < 0.05) intra-village spatial autocorrelation in index values in MG-1, KM-1, and MB-1, suggesting that some communities may have distinct spatial risk factor distributions (Fig 7). We observed that sub-communities, in particular caste or religious groups, tended to cluster together–but this clustering did not lead to discrete spatial domains with higher or lower risk as observed by our index. Villages in the same district tended to have similar risk index profiles, with significant differences among villages within districts only observed in Maharajganj (p = 0.005). This suggests that risk factors for aflatoxin contamination were evenly distributed within village communities, and that sub-populations were not localized in high-risk enclaves as has been observed for other public health threats, especially in larger cities [66]. The lack of spatial differentiation within communities likely reflected their small sizes, low populations, and generally low development status.

## Discussion

This study identified household-level factors associated with AFB1 contamination risk and indicated that risk factor profiles are specific to particular geographies. We developed and validated an index for predicting the likelihood of household AFB1 detection, which performed moderately well and could be used for formative risk assessment across spatial scales. To our knowledge, this is the first effort to establish a household-level AFB1 risk assessment system. Our findings reveal substantial predictive value of household characteristics for aflatoxin risk assessment, paving the way for future integration of household-level data into spatial surveillance systems.

Of the several risk factors identified *a priori* as potential drivers of aflatoxin contamination in these food systems, a household’s array of crop species was shown to be the most important determinant of risk status. Differential susceptibility of some commodities relative to others is driven to a great extent by crops’ host- or non-host compatibility with aflatoxigenic fungal pathogen, *Aspergillus flavus*, in a field setting. Maize and groundnuts, for example, are susceptible to *A*. *flavus* infection in the field, which translates into greater toxin loads both before and after harvest [67]. Other vulnerable commodities, such as rice and sorghum, can accumulate toxins both pre- and post-harvest [68, 69], but are not as vulnerable to *Aspergillus* molds in the field. Accordingly, cropping profiles can be used to gauge not only the level of aflatoxin contamination risk in a food system, but also the relative utilities of pre- versus post-harvest intervention options in a household or locality.

Across the four locations included in this survey, we observed marked variability in food system characteristics. The two northern locations, Maharajganj and Munger, were highly reliant on rice and wheat as dietary staples, and practiced rice (*kharif* season) and wheat (*rabi* season) crop rotations. Aflatoxin-susceptible commodities such as maize and groundnuts were present in both districts, but at low frequencies. Although these commodities were regularly consumed in the northern districts and constituted a substantial fraction of aflatoxin contaminated samples, they played relatively minor roles in local diets.

Kandhamal, in hilly southeastern India, had the least diverse food system and was largely dependent on a single growing season (*kharif*) of rice for subsistence. This reflects the Kandhamal villages’ lower socioeconomic status and relatively inhospitable growing environment. Owing to the rather sparse composition of local food systems, this was the only district whose aflatoxin burden was predominantly localized in rice. Higher aflatoxin detection rates and concentration levels in rice were observed in Kandhamal than in any other district. Mahabubnagar, situated in the semi-arid south, was wealthier (though still poor by global standards) and its food systems were more diverse. In this district, sorghum and groundnuts were common in local diets along with rice and wheat, which led to greater incidence of household-level aflatoxin detection in this district than the others. Maize cultivation and consumption were rare in this region. Mahabubnagar’s semi-arid climate did not permit crop cultivation during the summer cropping season.

The ranges of observed storage practices, grain sources, and dietary preferences varied markedly across the four districts. In India, smallholders’ grain storage practices are tightly bound to local knowledge and cultural traditions [70]. Moreover, it has been demonstrated that the various forms of storage containers (both traditional and conventional) have differential susceptibility to fungal contamination, as mediated by their microclimatic properties [71]. In this study, however, we did not detect significant differences in aflatoxin contamination among samples collected from different types of storage containers. A study of fungal contamination of sorghum from a range of village storage containers in the north Indian state of Punjab similarly concluded that despite the distinct properties of storage containers, fungal contamination and toxin deposition may be more influenced by crop variety, moisture levels, and other factors [71].

Grain sources (i.e. home-grown, market, etc.) can vary in relative mycotoxin contamination depending on the context [64, 72, 73]. We observed consistent aflatoxin levels across the range of grain sources (e.g. own farm, marketplace, PDS, etc.) reported by the smallholders, suggesting that source is not a major determinant of aflatoxin risk. We therefore hypothesize that growing conditions and the post-harvest management of grain are more substantial contributors to a household’s risk profile than their grain sources in the Indian context. This finding differs from what has been observed in African smallholder food systems, where there significant differences in aflatoxin levels between home-grown and market-derived grain have been observed [64, 73].

We observed a positive relationship between landholding and the likelihood of household aflatoxin detection in these food systems. This finding contradicts what has been observed in African smallholder contexts, where lower landholding size/socioeconomic status have been variably associated with higher aflatoxin biomarkers [74–77]. In the African communities studied, most smallholders consume a highly susceptible crop (maize) as a staple food, and therefore the negative relationship between socioeconomic status and aflatoxin exposure is attributable to poorer farmers’ inability to produce and preserve high-quality grain [75]. We hypothesized that India’s reliance on less susceptible commodities would result in an opposite relationship, as households with lower landholdings are less likely to consume highly susceptible commodities, such as maize, which are considered peripheral or specialty items in the local diet [78]. Consistent with this hypothesis, farmers with less land grew only rice and/or wheat, while those with more land were able to diversify their cropping systems to include commodities more susceptible to contamination than those local staples. The positive association between landholding and cropping diversity has been demonstrated previously in the Indian context but generally pertains only to smallholders, as Indian farmers with large (>2 ha) landholdings can choose to specialize in fewer crops grown in larger quantities [79, 80].

Food system composition, preventative behaviors, and farming versus non-farming status each had significant effects on household aflatoxin detection status in our prediction model. Household-level storage environmental parameters such as moisture content and relative humidity were not available in our data set, but incorporating these indicators into future iterations of our prediction model might enhance model performance. The presence/absence of groundnut in the household was an important determinant of contamination status, owing to the widespread distribution of this susceptible commodity within and across Indian food systems. Maize, despite its high susceptibility to aflatoxin contamination, was not predictive of detection status in this study, likely due to its overall rareness in Indian food systems. While contamination was frequently observed in other grain products, particularly sorghum and rice, the more sporadic distributions of contaminated samples made these crops less informative as risk predictors.

Agronomic practices and household food preservation behaviors can influence the initial level of fungal colonization of the storage environment and the magnitude of post-harvest aflatoxin contamination, respectively [3, 29, 30, 81]. While nine preventative behaviors were considered in initial model selection, just one (grain washing) had a significant reductive effect on the odds of household AFB1 detection. In addition to general hygiene, washing enables buoyancy-based density sorting, which has proven effective in mitigating aflatoxin levels in previous studies [82]. Among the households surveyed in this study, washing was generally practiced in tandem with hand sorting and drying, which have been shown to effect meaningful reductions in aflatoxin exposure [12, 30]. Therefore, it is likely that the observed importance of washing in the prediction model represents a combined effect of this suite of food safety behaviors.

We used several performance criteria and a repeated 5-fold cross-validation approach to determine the accuracy of the risk index in predicting household aflatoxin detection status. Practically, the score is easily calculable and can be immediately used to compare households and localities without the use of statistical models. The index classified aflatoxin contamination status with accuracy comparable to what has been achieved based on landscape-scale agroclimatic data alone. In one example from Australia, an aflatoxin risk index based on ambient temperature, radiation, rainfall, soil water and soil nitrogen predicted aflatoxin concentration with 69% accuracy [23]. In Europe, climate, radiation, and crop models predicted aflatoxin contamination in maize samples with 74–77% sensitivity and 23–65% specificity [83]. There remains substantial room for improvement in aflatoxin prediction modelling, both in our household-based model and in other agroclimatic approaches. The integration of household-level risk factors with data across scales, such as remotely sensed agroclimatic and edaphic characteristics, would likely achieve more accurate predictions than either set of variables could achieve independently.

Our novel household-level modeling approach elucidated risk factors that correspond to specific behaviors and decisions that can be targeted by intervention efforts. This feature is a major advantage of using household characteristics as the basis for risk assessment as opposed to local environmental conditions or sample-level biophysical properties, which cannot readily be targeted by behavior change programming in resource-poor settings. Our findings enabled the specific identification of vulnerable crops (i.e. groundnuts), important protective practices (i.e. post-harvest grain washing) and vulnerable sub-populations (i.e. non-farming households), all of which have been previously targeted in intervention pathways [14, 29, 84] and can be readily integrated into local diagnostic and problem-solving processes.

The relatively small sample size, limited geographical coverage, and unavailability of household-level pre-harvest risk factors (e.g. irrigation infrastructure, soil fertility management, etc.) in this preliminary study were likely constraints to prediction accuracy. Due to resource constraints and the exploratory nature of this study, we were unable to directly inform households exhibiting high toxin levels of their contamination status. However, the risk factor associations elucidated here have greatly informed downstream intervention work in similarly vulnerable communities. Another limitation of this study is that surveillance occurred during only one season; incorporating multi-seasonal data into the model would enhance the performance and utility of this approach. Despite these limitations, our findings serve as valuable evidence that household characteristics can be leveraged for aflatoxin risk assessment in smallholder farmer communities, with possible applications across diverse smallholder contexts. Given the high degree of variability in food system dynamics and sociocultural profiles across the developing world, we suspect that this modeling framework would reveal distinct risk factors if applied to contexts in Africa, Central America, or elsewhere in Asia. From this perspective, the odds ratio-based scoring system used in our study is ideal for cross-contextual application, as it would yield index values appropriately weighted to the risk profile of each environment.

The risk assessment system we present here is built on non-invasive, brief interactions with householders, and produces risk profiles that are readily interpretable and predictive of aflatoxin detection status. Given these features, local extension agents or other monitors could implement local risk analysis using this system, ideally validating the assessments for a subset of the samples. There are several existing programs in India that could benefit from these risk assessment tools. The government-sponsored *anganwadi* program, which is present in most villages and provides essential nutrition services for infants, children, and mothers, for example, has already been successfully leveraged for community-based cancer screenings [85]. This integration could serve as a model for localized screenings of community mycotoxin exposure risk. Moreover, >500,000 Village Health Sanitation and Nutrition Committees have served as important monitors of local health and nutrition and play vital roles in fostering connections with non-governmental organizations as implementation partners [86]. We envision that this aflatoxin risk assessment tool could be plugged into these collaborative efforts or other research programs to identify the breadth of aflatoxin risk factors within and across village communities and to set the stage for meaningful behavior change interventions.

## Supporting information

S1 Dataset Household survey data. Raw data generated during the household survey process in June-August, 2016 across the four participating districts.
(CSV)

S2 Dataset Sample aflatoxin B1 status and storage characteristics. (CSV)

S1 File Household survey questionnaire. The questionnaire was administered orally by enumerators in the respondents’ local language.
(PDF)
