# Supplementary material for: Exploring aflatoxin contamination and household-level exposure risk in diverse Indian food systems
Source: PLoS One. 2020 Oct 26;15(10):e0240565. doi: 10.1371/journal.pone.0240565 (PMC7588076; doi:10.1371/journal.pone.0240565)
Supplement: S1 File — The questionnaire was administered orally by enumerators in the respondents’ local language. (PDF) [file pone.0240565.s003.pdf]

Tata-Cornell Mycotoxin Survey

July-August 2016

Anthony Wenndt
ajw258@cornell.edu

Village:
Household Identifier Code:
Household Coordinates:
Oral Consent Received:

Part 1: Household Dynamics

1. Gender (Head of Household): M F OTHER
2. Landholding (acres, dismal, bigha, katha):
3. Type of Household (Circle most applicable):

NUCLEAR (PARENTS AND CHILDREN) EXTENDED/JOINT (3+ GENERATIONS)
SINGLE PARENT/WIDOW SINGLE ADULT

4. Number of household members:
5. Number of Children (0-14):
6. Who is responsible for food-related decisions? (Circle all applicable)

RESPONDENT SPOUSE CHILD OTHER

7. Who is responsible for farm-related decisions? (Circle all applicable)

RESPONDENT SPOUSE CHILD OTHER

8. Which best describes your farm?

SUBSISTENCE CASH CROP COMMERCIAL MIXED

9. What are your major Kharif crops?

10. What are your major Rabi crops?

11. What are your major summer crops?

Part 2: Food and Crop Management

1. How do you handle moldy grain (Circle all applicable)?

DISPOSE FEED TO LIVESTOCK SORT AND CONSUME OTHER

2. How do you ensure food safety (Circle all applicable)?

AVOID LONG STORAGE CHEMICAL ADDS. NATURAL ADDS. SUN DRYING
WASHING WASH AND SUN DRY SORT BY HAND OTHER

3. Describe your crop disease management strategy (Circle all applicable):

NO CONTROL PESTICIDES GOOD AGRONOMY RESISTANT VARIETIES
MANURE INPUT ORGANIC INPUT (FERTILIZER) OTHER

Part 3: Comments

1. Any supplemental comments or questions?

Part 2: Samples (Fill as many sheets as needed)

Sample #____

1. Sample Identifier (ex. VVHH01)[1]:
2. Crop Type:

RICE MAIZE WHEAT SORGHUM PULSES
SPICE (CHILLI, CORIANDER) OTHER

3. Crop Variety (if known):

LOCAL HYV HYBRID CERTIFIED SEED CROSS OTHER

4. Food Source

OWN FARM MARKET PDS NEIGHBOUR GIFT WAGES OTHER

5. Crop Form

WHOLE (MILLED) WHOLE (UNMILLED) ROUGH MEAL (COARSE)
SMOOTH MEAL (FINE) CHAFF/WASTE

6. Intended Use

CONSUMPTION LIVESTOCK FEED COMMERCIAL MIXED

7. Time in storage (days):
8. Storage Type

SACK PACKAGE TIN/PLASTIC CONTAINER PILE SILO/BIN OTHER

9. Disease Symptoms

NONE SOME DISCOLORATION (25%) MUCH DISCOLORATION (75%) MOLDY (100%)

Sample #____

1. Sample Identifier (ex. VVHH01):
2. Crop Type:

RICE MAIZE WHEAT SORGHUM PULSES
SPICE (CHILLI, CORIANDER) OTHER

3. Crop Variety (if known):

LOCAL HYV HYBRID CERTIFIED SEED CROSS OTHER

4. Food Source

OWN FARM MARKET PDS NEIGHBOUR GIFT WAGES OTHER

5. Crop Form

WHOLE (MILLED) WHOLE (UNMILLED) ROUGH MEAL (COARSE)
SMOOTH MEAL (FINE) CHAFF/WASTE

6. Intended Use

CONSUMPTION LIVESTOCK FEED COMMERCIAL MIXED

7. Time in storage (days):
8. Storage Type

SACK PACKAGE TIN/PLASTIC CONTAINER PILE SILO/BIN OTHER

9. Disease Symptoms

NONE SOME DISCOLORATION (25%) MUCH DISCOLORATION (75%) MOLDY (100%)

[1] VV = village code, HH = Household Identifier, 0X = sample number (01-05)
